# Decoding the enigmatic estrogen paradox in pulmonary hypertension: delving into estrogen metabolites and metabolic enzymes

**DOI:** 10.1186/s11658-024-00671-w

**Published:** 2024-12-18

**Authors:** Qiang You, Hequn Song, Ziming Zhu, Jinzheng Wang, Ruixin Wang, Mingjia Du, Yingjie Fu, Jinxiang Yuan, Rubin Tan

**Affiliations:** 1 Department of Physiology, Basic Medical School, Xuzhou Medical University, Xuzhou, 221004 Jiangsu China; 2 School of Pharmacy, Shandong University of Traditional Chinese Medicine, Jinan, 250355 Shandong China; 3 First Clinical Medical School, Xuzhou Medical University, Xuzhou, 221004 Jiangsu China; 4 College of Second Clinical Medical, Jining Medical University, Jining, 272067 Shandong China; 5 School of Nursing, Xuzhou Medical University, Xuzhou, 221004 Jiangsu China; 6 School of Pharmacy, Jining Medical University, Rizhao, 276826 Shandong China; 7 Lin He’s Academician Workstation of New Medicine and Clinical Translation, Jining Medical University, Jining, 272067 Shandong China

**Keywords:** Pulmonary hypertension, Estrogen, Hypoxia, Estrogen metabolites, CYPs, HSD17B

## Abstract

Pulmonary hypertension (PH) presents a puzzling sex bias, being more prevalent in women yet often less severe than in men, and the underlying reasons remain unclear. Studies using animal models, and limited clinical data have revealed a protective influence of exogenous estrogens, known as the estrogen paradox. Research suggests that beyond its receptor-mediated effects, estrogen acts through metabolites such as 2-ME2, 4-OHE2, and 16-OHE2, which are capable of exhibiting protective or detrimental effects in PH, prompting the need to explore their roles in PH to untangle sex differences and the estrogen paradox. Hypoxia disrupts the balance of estrogen metabolites by affecting the enzymes responsible for estrogen metabolism. Delving into the role of these metabolic enzymes not only illuminates the sex difference in PH but also provides a potential rationale for the estrogen paradox. This review delves into the intricate interplay between estrogen metabolites, metabolic enzymes, and PH, offering a deeper understanding of sex-specific differences and the perplexing estrogen paradox in the context of this condition.

**Graphic Abstract:**

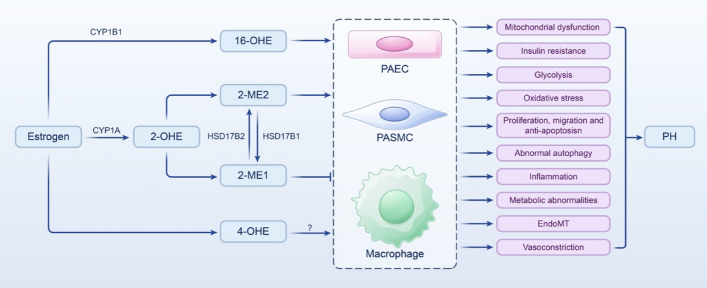

## Introduction

Pulmonary hypertension (PH) presents a complex diagnostic challenge, characterized by a resting mean pulmonary artery pressure exceeding the upper normal limit, typically 20 mmHg [1]. Across all age groups, from newborns to the elderly, PH can manifest due to various heart, lung, and systemic diseases, contributing to elevated morbidity and mortality rates [2]. Prolonged elevation of mean pulmonary artery pressure can precipitate hypoxia, ultimately leading to right heart failure and mortality, with no definitive cure currently available [3, 4]. The sixth World Symposium on Pulmonary Hypertension updated the clinical classification based on the World Health Organization PH groups, considering common pathological features, hemodynamics, and treatment approaches [5].

The two primary risk factors for PH are mutations in the bone morphogenetic protein receptor type II (BMPR2) gene and female gender [6, 7]. BMPR2 gene mutations are closely linked to the genetic form of PH. Women face a heightened risk of developing PH, with a male-to-female ratio of approximately 7:2 [8]. This significant female predisposition suggests an increased susceptibility to the disease [8, 9]. Despite the higher risk, women tend to exhibit less severe PH than men once diagnosed [7]. Notably, the prevalence of PH among perimenopausal women has surged, with the disease often presenting more severely compared with normal adult women [10, 11]. These observations underscore the potential influence of abnormal estrogen levels on the onset and progression of PH. Estradiol (E2) is the most abundant female sex hormone [12]. Although some studies have implicated E2 in promoting PH development [13], other studies have suggested that it has protective effects [14, 15]. This phenomenon highlights the estrogen paradox in PH, which the current research struggles to elucidate fully. In 2010, Tofovic et al. proposed a potential link between this paradox and estrogen metabolites [16].

This review delves deeper into the involvement of E2 and its metabolites in PH. The complexity of E2 metabolism can exert a potentially significant impact on the delicate balance between E2 and its metabolites and the pulmonary vascular environment; we explore the hypothesis that the relative proportion of estrogen metabolites may underlie why women are predisposed to PH but often experience milder forms of this condition. We aim to elucidate the mechanisms through which estrogen contributes to sex differences in PH and offer insights that may assist the development of novel prevention and treatment strategies.

## Estrogen metabolism

**Fig. 1:**
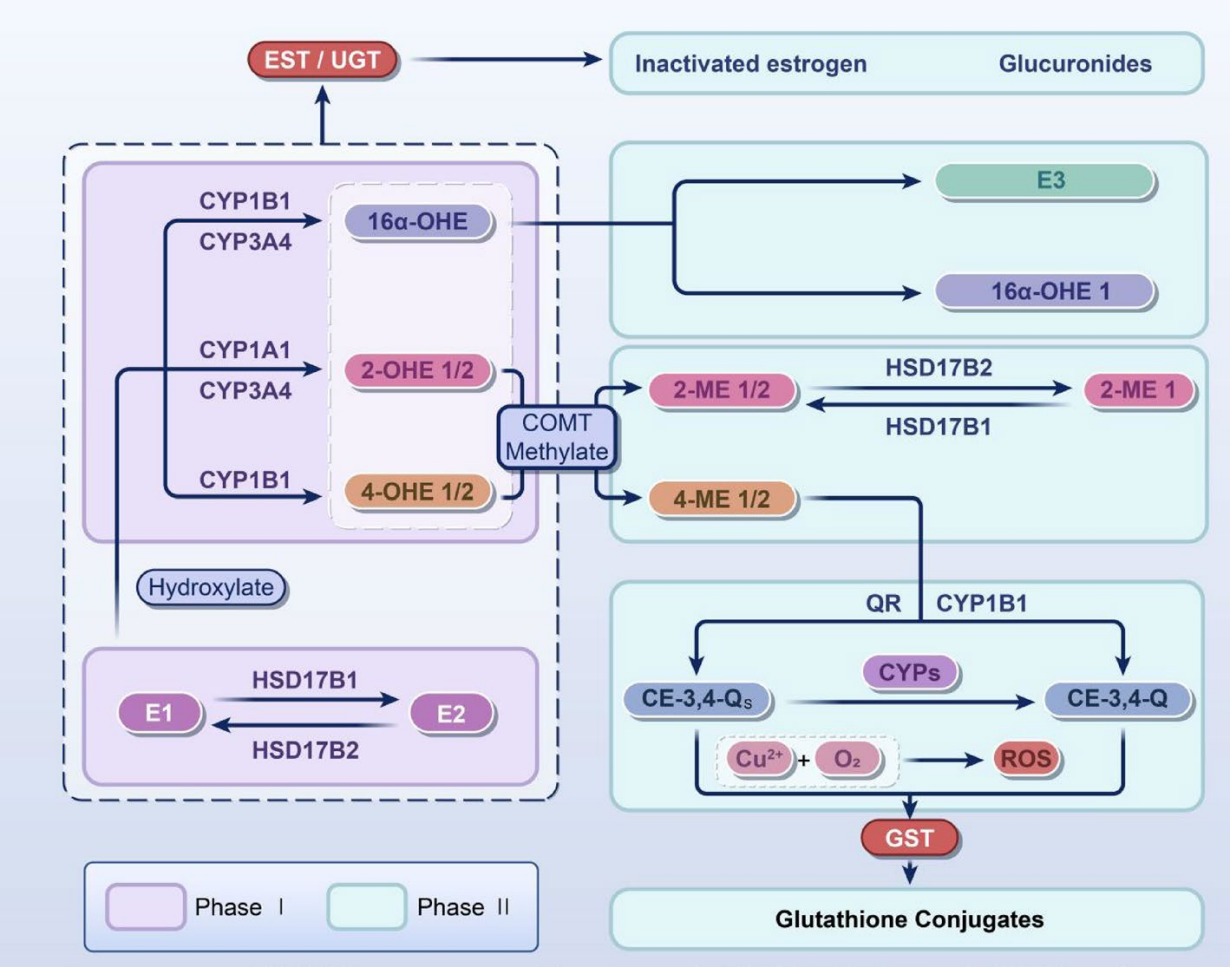
Main metabolic processes of estrogen. The main metabolic process of estrogen includes two stages. The first stage mainly forms metabolites dominated by 2-OHE, 4-OHE, and 16aα-OHE through CYPs. The second phase involves further metabolic processes of hydroxylated metabolites, including three major pathways: (1) methylation: COMT can convert 2-OHE and 4-OHE into 2-ME1/2 and 4-ME1/2; (2) glucuronidation: UGT/EST can convert estrogen or its hydroxylated metabolites into glucuronate; and (3) sulfonation: GST can convert these two quinone metabolites into less toxic small molecules, such as glutathione conjugates. E1, estrone; E2, estradiol; E3, estriol; EST, estrogen sulfotransferase; UGT, UDP-glucuronosyltransferase; 16α-OHE 1, 16α-hydroxyestrone; 16α-OHE, 16α-hydroxyestradiol; 2-OHE 1/2, 2-hydroxyestrone/2-hydroxyestradiol; 4-OHE 1/2, 4-hydroxyestrone/4-hydroxyestradiol; 2-ME2 1/2, 2-methoxyestrone/2-methoxyestradiol; 4-ME 1/2, 4-methoxyestrone/4-methoxyestradiol; QR, quinone reductase; CYPs, cytochrome p450s; CE-3:4-QS, catechol estrogen-3:4-semiquinone; CE-3:4-Q, catechol estrogen-3:4-benzoquinone; GST, glutathione *S*-transferase; COMT, catechol *O*-methyltransferase; HSD17B, 17β-hydroxysteroid dehydrogenase; ROS, reactive oxygen species. The arrows in the figure indicate transformations

Estrogen metabolism comprises two phases (Fig. 1): the first primarily involves oxidation, mainly hydroxylation, catalyzed by cytochrome P450s (CYPs) and the second encompasses three main reactions: *O*-methylation by catechol *O*-methyltransferase (COMT), sulfonation by sulfotransferases, and glucuronidation by UDP-glucuronosyltransferases (UGTs) [17–19].

### Phase I

Extensive research has suggested that the initial stage of estrogen metabolism commences with hydroxylation (Fig. 1). This process is catalyzed by phase I enzymes, particularly CYP1A1, CYP1B1, and CYP3A4, which hydroxylate E2 at different positions—2, 4, and 16 carbon sites—to produce 2-hydroxyestradiol (2-OHE2), 4-hydroxyestradiol (4-OHE2), and 16α-hydroxyestradiol, respectively [18]. Notably, CYP1A1 and CYP3A4 predominantly catalyze the production of 2-OHE2 in the liver, whereas CYP1B1 exhibits specific catalytic activity for 4-OHE2 in extrahepatic tissues [20]. 2-OHE2 is the main product of E2 metabolism, unlike estrone, whose main metabolite is 4-hydroxyestrone [21, 22].

### Phase II

Subsequently, these estrogen metabolites are inactivated by phase II enzymes, including UGT, glutathione *S*-transferase (GST), quinone reductase, sulfate transferases (SULTs), and COMT. These enzymes facilitate the coupling of oxidation and hydrolysis of compounds, achieving water solubility through *O*-methylation, sulfonation, or glucuronidation and rendering the final metabolites more readily excreted [23].

#### Estrogen *O*-methylation

The primary enzyme responsible for methylation is COMT, which facilitates the *O*-methylation of various endogenous substances containing catechol; COMT is crucial in the coupling and detoxification of diverse phase I metabolites [24] and catalyzing the methylation of 2-OHE2 to generate 2-methoxyestradiol (2-ME2), devoid of no estrogenic activity [25]. This *O*-methylation process serves as a pivotal physiological mechanism for the detoxification of 2-OHE2, safeguarding DNA molecules against oxidative stress by impeding their activity and the formation of mutagenic metabolites [26]. Subsequently, the generated 2-ME2 is converted by 17β-hydroxysteroid dehydrogenase (HSD17B) 2 to the less active 2-ME1 [27].

#### Estrogen sulphotransferase

Estrogen sulphotransferase (EST) is the principal metabolizing enzyme involved in sulfonation, a member of the SULT family participating in the inactivation of phase II metabolites [28]. Sulfonation is a coupling process involving the transfer of a sulfonate group from the sulfonate donor adenosine 3′-phosphate 5′-phosphate sulfate to the hydroxyl site of the acceptor molecule [29]. In addition, steroid sulfate esterase (STS), another member of the SULT family, exerts an opposing activity to EST, promoting estrogenic activity through desulfurization reactions and showing widespread presence and tissue specificity in the human body [30, 31]. In summary, EST and STS are vital in regulating phase II estrogen metabolites, crucial for maintaining human equilibrium.

#### Estrogen glucuronidation

The principal metabolic enzyme involved in glucuronidation is UGT, which converts estrogen and hydroxylated metabolites into water-soluble glucuronides, a key coupling reaction in the human body [32]. Studies by Zhu et al. revealed sex-based differences in UGTs between male and female rat livers, with the glucuronidation of estrone, E2, and 4-nitrophenol catalyzed by distinct UGTs occurring at a faster rate in females than in males [33]. Kallionpaa et al. showed that UGT1A10 was the most active enzyme in estrone glucuronidation, whereas UGT2B7 exhibited a higher rate of glucuronidation at the 16-OH position [34].

In summary, many animal experiments have shown that most estrogen-metabolizing enzymes show tissue- and sex-specific differences in their expression. Moreover, these metabolic enzymes may respond differently to changes in estrogen levels in women, potentially inducing diseases. These findings are consistent with the higher prevalence of PH in women, although determining the underlying mechanisms requires further investigation. Recently, James et al. identified a novel metabolite of estrone, preliminarily identified as stable 5α,6α-epoxyestrone, which may exert effects similar to catechol estrogens and also display genetic toxicity [35]. The functions of specific estrogen metabolites and their relationship with PH are elucidated below.

## The role of estrogen metabolites in PH

### 16α-Hydroxyestrone (16α-OHE) in PH

**Fig. 2:**
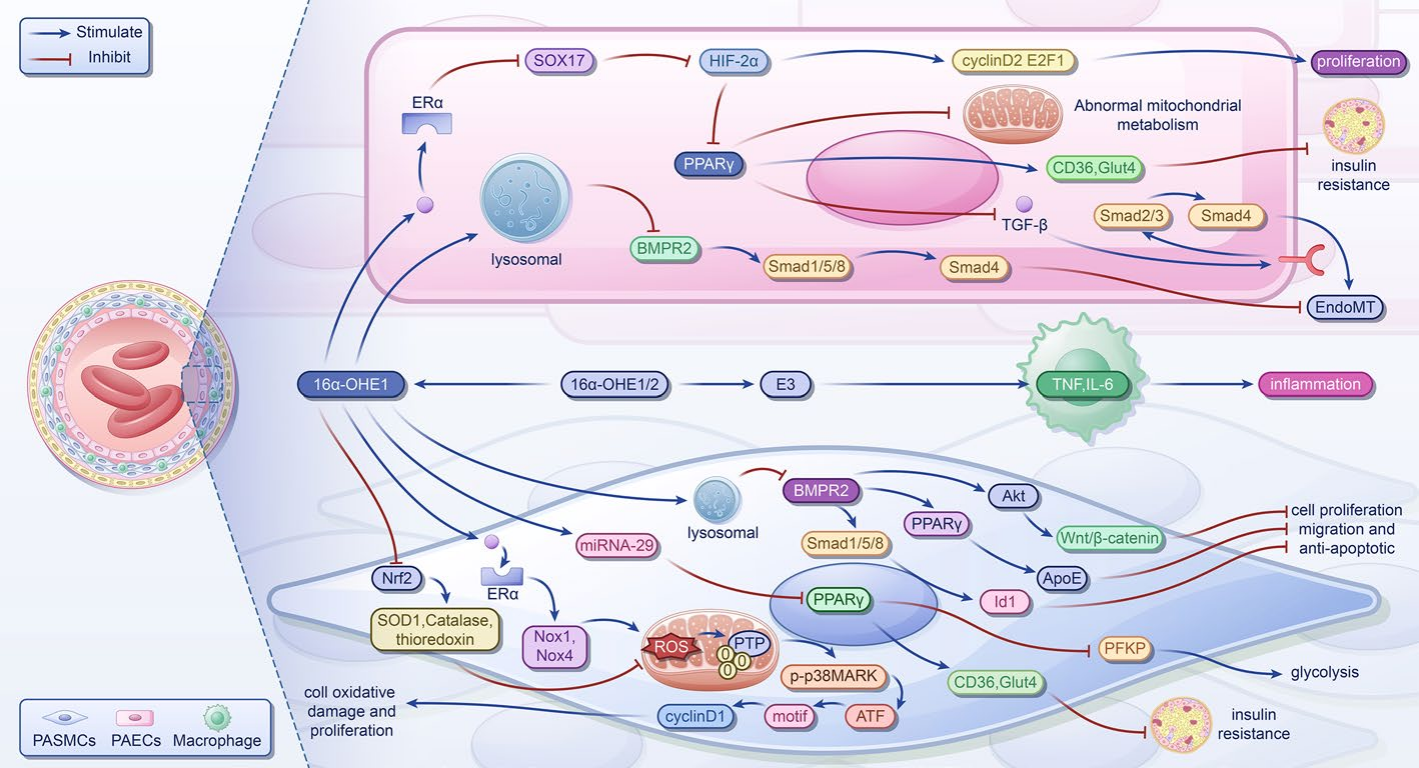
The mechanism of 16α-OHE promoting pulmonary hypertension. In PASMCs, 16α-OHE1 binds with ERα to inhibit Nrf2, resulting in upregulation of Nox1 and Nox4, decreased antioxidants increased ROS production, and irreversible PTP oxidation. Activation of the p38MARK pathway leads to increased phosphorylation of CRE region and ATF-2, upregulation of cyclinD1, and promotion of cell oxidative damage and proliferation. Upregulation of miRNA-29 inhibits expression of PPARγ, further reducing CD36 and Glut4 and upregulating PFKP, resulting in insulin resistance and increased aerobic glycolysis of 16α-OHE1, which may promote cell, migration, and antiapoptosis by reducing BMPR2 levels and inhibiting the BMPR2-Smad1/5/8-ID1, BMPR2-AkT-Wnt/β-Catenin, and BMPR2-PPARγ-apoe signaling pathways through lysosomal activation. In PAECs, 16α-OHE1 binds with Erα to inhibit X17, upregulate HIF-2α, and increases cyclin D2 and E2F1, promoting cell proliferation. Additionally, 16α-OHE1 inhibits PPARγ and attenuates mitochondrial bioenergy and insulin resistance, leading to metabolic abnormalities. Inhibition of PPARγ and BMPR2 promotes EndoMT through inhibition of p-Smad1/5/8-Smad4 signaling and enhancement of TGF-β-Smad2/3-Smad4 signaling. E3 stimulates the expression of TNF and IL-6, potentially exerting a proinflammatory effect in PH. E3, estriol; 16α-OHE1/2, 16α-hydroxyestrone/16α-hydroxyestradiol; TNF, tumor necrosis factor; IL-6, interleukin-6; Nrf2, nuclear factor E2-related factor 2; SOD1, superoxide dismutase 1; ROS, reactive oxygen species; Nox1/4, nicotinamide adenine dinucleotide phosphate oxidase1/4; PTP, protein tyrosine phosphatases; p38MARK, p38 mitogen-activated protein kinase; CRE, cAMP response element; ATF-2, activating transcription factor 2; ERs, estrogen receptors; SOX17, SRY-related HMG-box 17; HIF-2α, hypoxia-inducible factor 2α; E2F1, E2F transcription factor 1; PPARγ, peroxisome proliferator-activated receptor γ; CD36, cluster of differentiation 36; Glut4, glucose transporters type 4; PFKP, platelet-type phosphofructokinase; BMPR2, bone morphogenetic protein receptor type II; Id1, DNA binding 1; Akt, protein kinase B; apoE, apolipoprotein E; TGF-β, transforming growth factor-β; EndoMT, endothelial-to-mesenchymal transition; PASMC, pulmonary artery smooth muscle cell; PAEC, pulmonary arterial endothelial cell

A metabolite of E2, known as 16α-OHE, exhibits potent estrogenic activity [36]. Extensive research has demonstrated that 16α-OHE can modulate the cell cycle by covalently binding to estrogen receptor (ER) α, exerting genotoxic effects, and fostering the progression of various diseases [37, 38]. In experimental PH, 16α-OHE has been implicated in the disease process by stimulating cell proliferation [39]. Furthermore, under modifying factors such as obesity, the heightened production of 16α-OHE through metabolism can induce oxidative damage in cells, contributing to the development of PH [40]. Additionally, studies have indicated that 16α-OHE exacerbates dysregulation in pathways associated with vascular injury, including angiogenesis, insulin resistance, and the Wnt signaling pathway [41]. Consequently, we delve into the molecular mechanisms through which 16α-OHE promotes PH, encompassing induction of oxidative stress, enhancement of proliferation and migration of pulmonary arterial smooth muscle cells (PASMCs) and pulmonary arterial endothelial cells (PAECs), promotion of metabolic abnormalities, induction of endothelial mesenchymal transition (EndoMT), and fostering inflammation (Fig. 2).

#### 16α-OHE inducts oxidative stress

Experimental evidence indicates that 16α-OHE contributes to cell damage and excessive proliferation of PASMCs by inducing oxidative stress, thereby participating in PH [42]. On one hand, 16α-OHE activates ERα, leading to upregulation of nicotinamide adenine dinucleotide phosphate oxidase (Nox)1 and Nox4 expression and increases reactive oxygen species (ROS) production, mainly superoxide anion and hydrogen peroxide, mediated by these enzymes. On the other hand, 16α-OHE suppresses the antioxidant effect of nuclear factor E2-related factor 2 (Nrf2), reducing downstream basal levels of antioxidants such as superoxide dismutase (SOD) 1, catalase, and thioredoxin [42, 43]. Excessive ROS production can lead to the irreversible oxidation of PTP and activation of p38 mitogen-activated protein kinase (p38MAPK). The proliferation of PASMC is illustrated by an increase in PCNA and a decrease in the expression of CDK inhibitor p27 [42, 44].

#### 16α-OHE promotes cell proliferation and migration

Moreover, 16α-OHE exhibits potent effects on proliferation and migration. It activates p38MAPK [42], further enhancing the activation of the CRE region of the cyclinD1 promoter and the phosphorylation of ATF-2. This leads to increased cyclinD1 expression, which promotes cell growth and cycle progression [45–47]. Additionally, Fessel et al. demonstrated that 16α-OHE could increase the PH permeability of BMPR2 mutant mice, while in control mice, it downregulated the BMPR2 protein level and inhibited its signaling pathway [41]. This effect may be regulated by 16α-OHE-mediated activation of lysosomes in a nontranscriptional manner [41, 48]. In PASMCs, this can further diminish canonical and noncanonical BMPR2 signaling pathways, such as BMPR2-Smad1/5/8-Id1, BMPR2-protein kinase B (Akt)-Wnt/β-catenin, and BMPR2-peroxisome proliferator-activated receptor γ (PPARγ)-apoE, promoting cell proliferation, migration, and antiapoptosis [49–51]. In PAECs, BMPR2 deficiency enables cells to achieve contradictory phenotypes, apoptosis, and proliferation, though the mechanisms driving these processes in vivo remain unclear [52, 53].

#### 16α-OHE promotes metabolic abnormalities

Metabolic dysfunction has emerged as a significant pathological feature of PH and is closely associated with structural and functional mitochondrial abnormalities. Notably, PH-related metabolic disturbances predominantly involve a shift from oxidative phosphorylation to aerobic glycolysis—known as the Warburg effect—and systemic insulin resistance. In experiments utilizing a BMPR2 gene mutant mouse model, it was discovered that 16α-OHE upregulates miRNA-29, leading to reduced PPARγ expression, subsequently diminishing the levels of CD36 and Glut4, thereby impairing insulin mobilization and inducing insulin resistance [54, 55]. Concomitantly, decreased PPARγ levels can elevate platelet-type phosphofructokinase (PFKP) activity, further promoting glycolysis [56]. In PASMCs, diminished oxidative phosphorylation reduced acetyl-CoA production. According to the Randles cycle, decreased acetyl-CoA levels activate CPT1 expression, thereby upregulating fatty acid oxidation, increasing cellular ATP levels, inhibiting AMPK activation, and fostering cell proliferation [57]. Additionally, recent investigations uncovered that 16α-OHE, via ERα signaling in PAECs, suppresses SRY-related HMG-box 17 (SOX17), exacerbating PH [58]. SOX17 downregulation attenuates hypoxia-inducible factor (HIF)-2α inhibition, precipitating metabolic dysregulation [58]. This effect may be achieved by further suppressing PPARγ expression, enhancing carnitine shuttle capacity, and dampening mitochondrial bioenergetics [59, 60]. Furthermore, HIF-2α can boost cyclin D2 and E2F1 expression through c-Myc mediation, thereby promoting cell proliferation [61].

#### 16α-OHE inducts EndoMT

EndoMT represents a critical pathway in pulmonary vascular remodeling observed in PH and has been documented in various models, including systemic sclerosis (SSc)-associated pulmonary arterial hypertension (PAH) and SU-5416/hypoxia-induced PH [62–64]. EndoMT involves a phenotypic shift of endothelial cells (ECs) toward a myofibroblast or mesenchymal phenotype [62–64]. The process is primarily triggered by increased transforming growth factor-β (TGF-β) and decreased BMPR2 signaling. In conditions of mechanical stress, inflammation, oxidative stress, and hypoxia, 16α-OHE reduces PPARγ levels, thus relieving the inhibitory effect on Smad3 and enhancing TGF-β-Smad 2/3-Smad4 signaling, thereby promoting EndoMT [54, 56]. Furthermore, 16α-OHE downregulates BMPR2 protein levels, potentially further activating EndoMT by alleviating the inhibitory effect of the BMPR2-Smad1/5/8-Smad4 signaling pathway [41].

#### 16α-OHE promotes inflammation

Moreover, Fessel et al. demonstrated the anticipated protective role of 16α-OHE against classical cytokine-induced inflammation [41]. Noteworthy cytokine alterations observed in BMPR2 mutants and control animals include the downregulation of Ccl3, CSF3R, interleukin (IL)-1β, IL-8, LILRB4, and lectin. Conversely, it increases genes associated with vascular injury or angiogenesis, such as platelet glycoproteins GP5 and GP9, suggesting that 16α-OHE may induce inflammation through noncytokine pathways, although the precise mechanism remains unclear [41]. Additionally, another metabolite, E3, produced through 16-hydroxylation, has been found to strongly stimulate the expression of tumor necrosis factor (TNF) and IL-6, implying its potential proinflammatory role in PH [65].

### 2-ME2 in PH

**Fig. 3:**
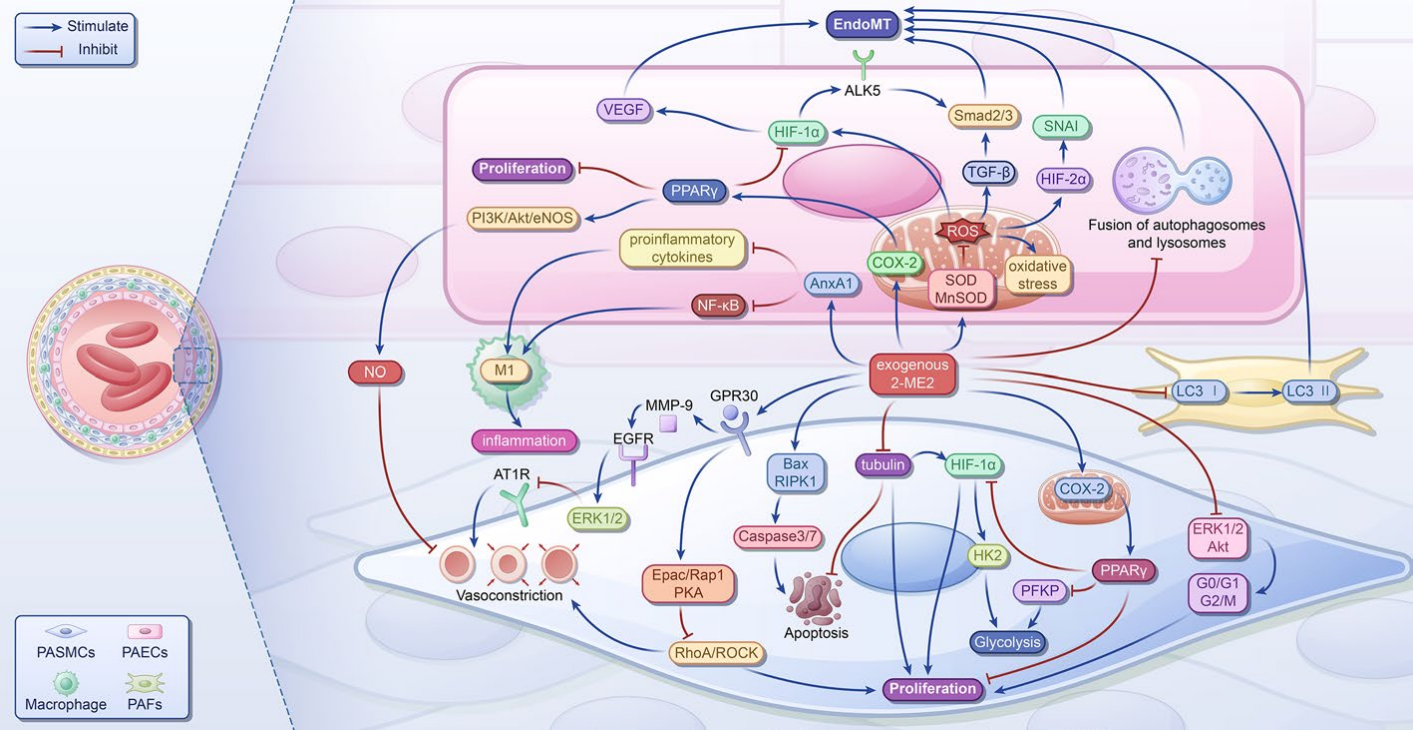
The mechanism of 2-ME2 suppressing pulmonary hypertension. In PASMCs, 2-ME2 indirectly activates PPARγ by promoting COX-2 expression, inhibiting proliferation. It also binds GPR30, activating Epac/Rap1 and PKA, inhibiting RhoA/ROCK expression, and, ultimately, inhibiting proliferation and vasoconstriction. In addition, 2-ME2 inhibits the activation of ERK1/2/Akt, blocking the G0/G1 and G2/M phase of the cell cycle and inhibiting proliferation. It disrupts tubulin, promotes apoptosis, and activates PPARγ, inhibiting PFKP and HIF-1α/HK2 to inhibit glycolysis. Furthermore, 2-ME2 binds GPR30, transactivating EGFR by releasing MMP-9, to activate ERK1/2, inhibit AT1R expression, and inhibit vasoconstriction. In pulmonary artery endothelial cells (PAECs), 2-ME2 activates PPARγ, which may inhibit proliferation and activate PI3K/Akt/eNOS, increase NO release, and inhibit vasoconstriction. It also inhibits HIF-1α, VEGF, and TGF-β/Smad2/3 to inhibit EndoMT; 2-ME2 inhibits SOD and MnSOD to inhibit ROS and oxidative stress, inhibiting HIF1α, TGF-β, and HIF-2α/SNAI, ultimately inhibiting EndoMT. It also inhibits the fusion of autophagosomes and lysosomes to inhibit EndoMT. Additionally, 2-ME2 promotes AnxA1 expression, inhibiting proinflammatory cytokines and NF-κB and inhibiting inflammation; 2-ME2 inhibits macrophage activation, inflammation, and early LC3 transformation in PAFs to inhibit EndoMT. PPARγ, peroxisome proliferator-activated receptor γ; 2-ME2, 2-methoxyestradiol; Bax, Bcl-2-associated X protein; COX-2, cyclooxygenase-2; PFKP, platelet-type phosphofructokinase; HIF-1α, hypoxia-inducible factor-1α; HIF-2α, hypoxia-inducible factor-2α; HK2, hexokinase 2; RhoA, Ras homolog gene family member A; ROCK, Rho-kinase; ERK1/2, extracellular signal-regulated kinases1/2; GPR30, G protein-coupled receptor 30; Epac, exchange protein activated by cAMP; Rap1, Ras-associated protein 1; PKA, protein kinase A; Akt, protein kinase B; MMP-9, matrix metalloproteinase-9; EGFR, epidermal growth factor receptor; AT1R, angiotensin II type I receptor; PI3K, phosphatidylinositol 3-kinase; eNOS, endothelial nitric oxide synthase; NO, nitric oxide; VEGF, vascular endothelial-derived growth factor; ALK5, activin receptor‑like kinase-5; RIPK1, receptor-interacting protein kinase 1; SOD, superoxide dismutase; MnSOD, manganese superoxide dismutase; ROS, reactive oxygen species; TGF-β, transforming growth factor-β; SNAI, zinc finger protein SNAI family; AnxA1, annexin A1; NF-κB, nuclear factor-kappa B; LC3, light chain 3; EndoMT, endothelial-to-mesenchymal transition; PASMC, pulmonary artery smooth muscle cells; PAEC, pulmonary arterial endothelial cell

2-ME2 exhibits a low affinity for classical ERα and ERβ nuclear receptors and displays vasoprotective properties [66–68]. Several studies have underscored the efficacy of 2-ME2 in inhibiting PAH progression primarily by impeding abnormal cell proliferation and mitigating aberrant mitochondrial function [69, 70] (Fig. 3). Additionally, 2-ME2 impedes vascular EndoMT through various pathways (Fig. 3), potentially yielding similar benefits in PAH [71–73]. Furthermore, several studies have revealed the protective effects of 2-ME2 against inflammation-related ailments (Fig. 3), suggesting its potential impact on the development of PH [74–76].

#### 2-ME2 suppresses proliferation

2-ME2 suppresses smooth muscle cells (SMCs) by inhibiting proliferation and promoting apoptosis. Research conducted on human aortic smooth muscle cells (HASMCs) has demonstrated that the antiproliferative effect of 2-ME2 primarily involves impeding the initiation and progression of the mitotic program and cell division [71, 77–80]. By targeting the extracellular signal-regulated kinase (ERK) 1/2 and Akt signaling pathways, 2-ME2 inhibits the serum-stimulated G0/G1 and G2/M phases of the cell cycle in HASMCs, thereby preventing mitotic initiation and inducing apoptosis in arrested cells [71, 78, 79]. Furthermore, 2-ME2 disrupts tubulin organization in HASMCs by binding to colchicine sites, thereby impeding mitosis and suppressing HIF-1α activity, which further inhibits cell proliferation [71, 77, 79, 80]. In PDGF-BB-induced HASMCs, 2-ME2 inhibited the Ras homolog gene family member A (RhoA)/Rho-kinase (ROCK) 1 pathway by downregulating the mRNA expression of genes and suppressing cell division [79]. Moreover, in severe PAH, decreased expression levels of the PPARγ gene and protein lead to abnormal growth of endothelial and vascular SMCs [81–83]; 2-ME2 promotes the activation of PPAR-related genes in human PASMCs by upregulating cyclooxygenase-2 (COX-2) expression, thereby mitigating abnormal growth [84]. Additionally, 2-ME2 disrupts protein transport in cells by targeting α-tubulin, inducing proapoptotic effects on human PASMCs [77]. It also enhances caspase3/7 activity by upregulating the transcription of Bax and RIPK1, which ultimately leads to apoptosis [77]. Activation of PPARγ by 2-ME2 may also contribute to inhibiting abnormal ECs growth in PH.

#### 2-ME2 promotes vascular relaxation

G protein-coupled receptor 30 (GPR30) is a high-affinity membrane receptor for 2-ME2 [73], activating-mediating vasodilatory effects. In porcine coronary artery SMCs, GPR30 activation triggers RhoA/ROCK inactivation by stimulating Epac/Rap1 and protein kinase A (PKA), leading to coronary artery relaxation [85]. In rat aortic SMCs and HASMCs, 2-ME2 activates GPR30 and subsequently transactivates EGFR by releasing matrix metalloproteinase-9 [86]. This cascade activates ERK1/2 and ultimately downregulates AT1R mRNA expression, relieving Ang II-induced vasoconstriction by reducing intracellular calcium release [73, 86]. The binding of 2-ME2 to GPR30 may also mediate vasodilation in PAH. Gui et al. discovered that 2-ME2 pretreatment stimulates protein synthesis linked to the endothelial nitric oxide synthase (eNOS) pathway within ECs, independent of ER or GPR30 [87]. This leads to increased nitric oxide (NO) production, which acts on vascular SMCs to reduce light chain (LC) 20 phosphorylation, ultimately inhibiting the contraction of rat aortic rings induced by phenylephrine stimulation [87]. Furthermore, experiments conducted with human umbilical vein ECs and rat aorta have shown that 2-ME2 enhances NO release, inducing vasodilation by indirectly activating PPARγ [88]. This activation triggers the phosphatidylinositol 3-kinase (PI3K)/Akt/eNOS cascade in vascular ECs [88]. Additionally, 2-ME2 contributes to vascular relaxation by promoting prostaglandin production by upregulating COX-2 gene expression [71].

#### 2-ME2 suppresses the mitochondrial abnormalities

Mitochondrial dysfunction is a key feature of PAH and is characterized by a metabolic shift from glucose oxidation to uncoupled aerobic glycolysis, known as the “Warburg metabolic transition” [89]. This metabolic reprogramming is evident in PASMCs, PAECs, epithelial fibroblasts, and right ventricular cardiomyocytes from patients with PAH, promoting cell proliferation and evading mitochondrial apoptosis [90–94]. In PAH, TGF-β1 is upregulated, particularly in PASMCs, with a stronger stimulatory effect observed in PAH PASMCs than in patients with chronic obstructive pulmonary disease [56, 95, 96]. TGF-β1 stimulation in small and medium pulmonary arteries of idiopathic patients with PAH and cultured PAH PASMCs induces mitochondrial activation and upregulates PFKP expression, a key enzyme in promoting proliferation and preglycolysis [56]. This process is reversed by the activation of PPARγ [56]. Since 2-ME2 activates PPARγ, it may inhibit glucose metabolism and the progression of PAH through this pathway. In human PASMCs, 2-ME2 inhibits HIF-1α through PPARγ, downregulating HK2 and inhibiting proliferation [77]. Mitochondrial dysfunction in PAH leads to increased ROS production, elevated oxidative stress, and metabolic reprogramming [97]. Reduced expression of manganese superoxide dismutase (MnSOD) in PAH ECs results in ROS accumulation and decreased NO expression, leading to increased HIF-1 expression [98]. Knockdown of HIF-1 via miRNA increases mitochondrial numbers in PAECs [98]; 2-ME2 reduces ROS production by inhibiting the HIF-1α/Nox-related pathway, inhibiting vascular remodeling, and mitigating PAH in chronically hypoxic-induced rats [69]. Additionally, 2-ME2 treatment improves mitochondrial ultrastructural damage, reduces ROS levels, increases SOD activity, and enhances MnSOD activity and expression, thereby reducing HIF-1α transcription and translation, ultimately inhibiting pulmonary vascular remodeling and HPH development [70].

#### 2-ME2 suppresses EndoMT

In human PAECs, 2-ME2 exhibits inhibitory effects on the increased expression of ALK5 by suppressing HIF-1α [72, 99]. This inhibition leads to the downregulation of Smad2/3 phosphorylation, ultimately impeding hypoxia- and radiation-induced EndoMT and pulmonary fibrosis, as confirmed by experiments using human umbilical vein ECs and mouse models [72, 99]. Moreover, 2-ME2 mitigated collagen synthesis and EndoMT in hypoxia-induced scleroderma by impeding the early transformation of fibroblasts and inhibiting autophagosome-lysosome fusion in human umbilical vein ECs [100]. The HIF-1α/vascular endothelial-derived growth factor (VEGF) signaling pathway plays an important role in hypoxia-induced EndoMT of human microvascular ECs [101], and 2-ME2 potentially inhibits EndoMT by suppressing this pathway. Elevated levels of HIF-2α induced by PHD2 downregulation trigger EndoMT via the upregulation of SNAI in lung vascular ECs from patients with idiopathic PAH and monocrotaline-induced PH rats [102]. This process contributes to the thickening of the cardiopulmonary vascular wall and the development of occlusive pulmonary vascular intimal lesions [102]. Hypoxia-induced ROS stabilize HIF subunits (including HIF-2α) and activate EndoMT by inducing endogenous expression of TGF-β and activating latent TGF-β [64, 103]; 2-ME2 exerts inhibitory effects on EndoMT by potentially inhibiting ROS.

#### 2-ME2 suppresses inflammation

Clinical evidence indicates that persistent chronic inflammation is a hallmark of PAH that contributes to disease progression [104]. Patients with PAH exhibit elevated serum levels of various inflammatory markers linked to disease severity and patient survival, including IL-1b, IL-2, IL-4, IL-6, IL-8, IL-10, IL-12, IL-18, TNF, CRP, and MCP-1 [105–109]; 2-ME2 has demonstrated protective effects against inflammation-related diseases such as rheumatoid arthritis and experimental autoimmune encephalomyelitis [74, 75]. In models of ischemia–reperfusion-induced acute pneumonia, 2-ME2 inhibits nuclear factor-kappa B (NF-κB) activation and reduces levels of proinflammatory cytokines such as IL-6, CINC-1, and TNF by upregulating the expression of AnxA1, ultimately ameliorating inflammation [110]. Furthermore, 2-ME2 treatment decreases transendothelial migration of hypoxia–reoxygenation-exposed neutrophils in vitro, reduces TNF production, and enhances apoptosis [110]. Additionally, 2-ME2 demonstrated protective effects against renal ischemia–reperfusion damage by attenuating NF-κB activity and proinflammatory cytokine expression [111]. Both in vitro and in vivo, the anti-inflammatory properties of 2-ME2 are partly mediated by the inhibition of macrophage activation [76].

### 4-OHE2 in PH

**Fig. 4:**
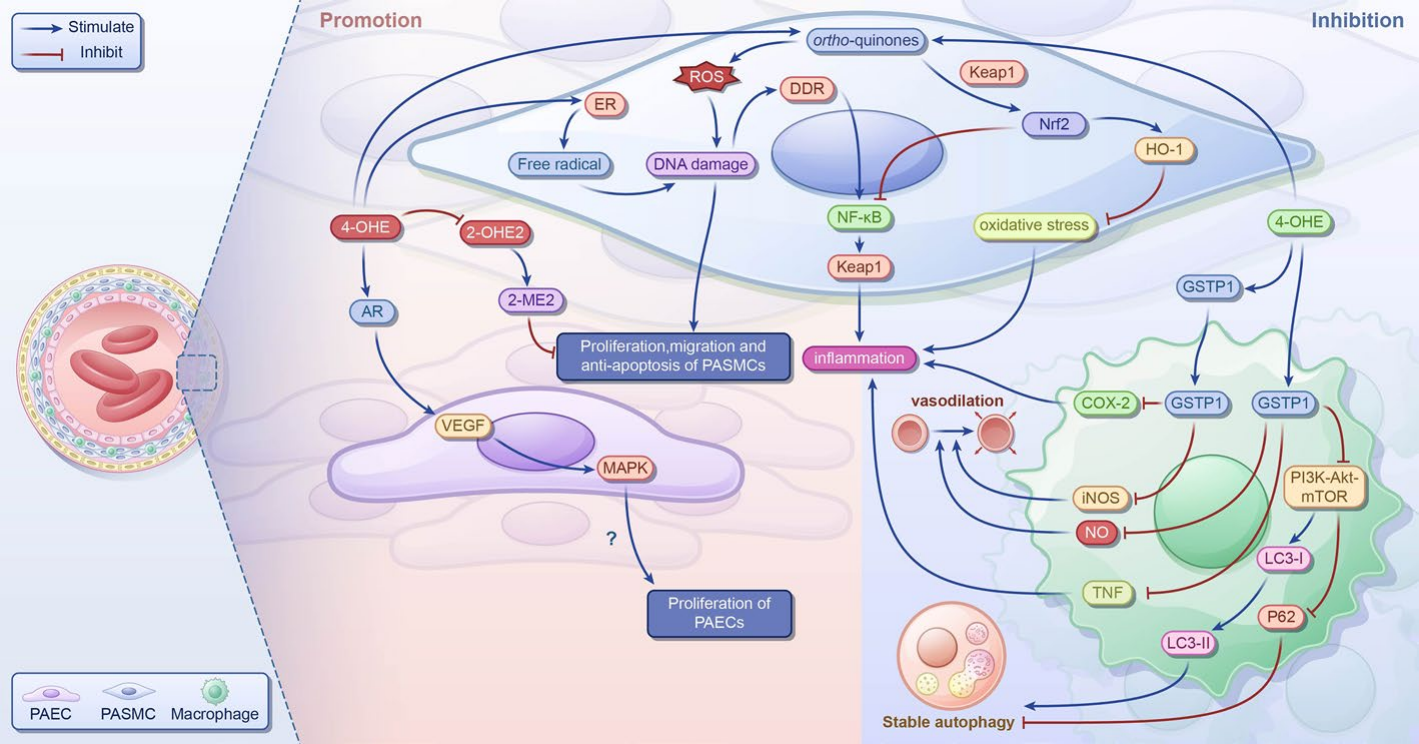
The role of 4-OHE in pulmonary hypertension; 4-OHEs competitively decrease the activity of 2-OHE2, inhibiting the formation of 2-ME2. They activate ERs, leading to the production of free radicals and generation of *ortho*-quinones, resulting in ROS production and DNA damage, which can cause dysfunction of PAECs and promote the proliferation and migration of PASMCs. By binding to ARs, 4-OHEs increase VEGF expression and activate ERK1/2, JNK, and p38MAPK pathways and promote the proliferation of AECs; 4-OHEs induce DDR and upregulate the levels of proinflammatory cytokines IL-1β, IL-6, and TNF, potentially aggravating the deterioration of PH. However, 4-OHEs also increase HO-1 levels through the Nrf2–Keap1–ARE pathway, inhibiting oxidative stress and PASMCs proliferation and promoting apoptosis. In lung tissue, they enhance Nrf2 expression, reducing the levels of proinflammatory cytokines IL-8 and TNF. In LPS-stimulated macrophages, 4-OHEs upregulate GSTP1, decreasing the levels proinflammatory cytokines TNF, NO, iNOS, and COX-2, mitigating inflammation, and potentially ameliorating PH deterioration. Furthermore, they inhibit the PI3K–Akt–mTOR pathway and LC3-II levels, decrease p62 levels, and enhance autophagy stability. 4-OHEs, 4-hydroxyestrogens; 2-OHE2, 2-hydroxyestradiol; 2-ME2, 2-methoxyestradiol; ERs, estrogen receptors; ARs, adrenergic receptors; VEGF, vascular endothelial-derived growth factor; ERK1/2, extracellular signal-regulated kinases1/2; JNK, c-Jun N-terminal kinase; p38MAPK, p38 mitogen-activated protein kinase; AECs, arterial endothelial cells; DDR, DNA damage response; IL, interleukin; TNF, tumor necrosis factor; HO-1, heme oxygenase 1; Nrf2, nuclear factor E2-related factor 2; Keap1, Kelch-like ECH-associated protein 1; ARE, antioxidant response element; LPS, lipopolysaccharide; COX-2, cyclooxygenase-2; GSTP1, glutathione *S*-transferase P1; NO, nitric oxide; iNOS, inducible nitric oxide synthase; PI3K, phosphatidylinositol 3-kinase; Akt, protein kinase B; mTOR, mechanistic target of rapamycin; LC3-II, light chain 3-II

In PH, 4-OHE2 is the product of CYP1B1 hydroxylation at the 4-carbon position of E2 [112]; 2-ME2 has protective effects such as antimitotic, antiproliferative, antiangiogenic, and anti-inflammatory effects [79, 113]. Research indicates that 4-OHE2 inhibits the binding of E2 to ERs and competitively reduces the activity of 2-OHE2 [114], potentially hindering the production of protective 2-ME2. This suggests that 4-OHE2 might exacerbate PH progression (Fig. 4). Furthermore, studies have highlighted the role of 4-OHE2 in promoting cell and tissue proliferation [115], which could adversely affect the development of PH. However, the precise mechanism linking 4-OHE2 to PH remains unexplored; some studies have suggested that 4-OHE2 might alleviate PH progression through related mechanisms (Fig. 4).

#### 4-OHE2 exacerbates PH

Previous studies have suggested that 4-OHE2 may exacerbate PH by stimulating cell proliferation; 4-OHE2 produces free radicals by activating ERs [116]. In addition, it produces ROS via oxidation to form *ortho*-quinone, both of which cause DNA damage [116, 117]. DNA damage can cause pulmonary ECs dysfunction and promote the proliferation and migration of pulmonary vascular smooth muscle [118]. Additionally, 4-OHE2 binds to adrenergic receptors and triggers the activation of ERK1/2, c-Jun N-terminal kinase, and p38MAPK pathways via VEGF, promoting the proliferation of uterine artery ECs in pregnant ewes [119]. However, whether similar effects occur in the pulmonary artery remains unclear.

Notably, DNA damage induced by 4-OHE triggers the activation of the NF-κB pathway via the DNA damage response, leading to the upregulation of inflammatory factors such as IL-1β, IL-6, and TNF, thereby inciting an inflammatory response [120]. Inflammation is a pivotal pathological feature of patients with PH, and its excessive activation can promote PH progression [121].

#### 4-OHE2 ameliorate PH

Conversely, 4-OHE2 may ameliorate PH by inhibiting cell proliferation via distinct mechanisms. Through redox cycling, 4-OHE2 generates an electrophilic quinone, which promotes Nrf2 activation by covalently binding to Kelch-like ECH-associated protein 1 (Keap1) [122]. In a rat model of hypoxia-induced PH, activated Nrf2 enhanced HO-1 expression via the Nrf2–Keap1–ARE signaling pathway, exerting antioxidative stress effects, inhibiting PASMCs proliferation, and promoting apoptosis, thereby mitigating PH development [119, 123]. Within lung tissue, Nrf2 inhibits NF-κB activation, thereby reducing IL-8 and TNF expression and curbing inflammation [124].

In addition, 4-OHE is an effective irreversible GST inactivation agent [125]. Among glutathione *S*-transferase P (GSTP) isozymes, GSTP1 exhibits antioxidant and anti-inflammatory effects [126]. Bin Xue et al. found that in lipopolysaccharide (LPS)-stimulated macrophage-like cells, the expression of GSTP1 was upregulated, which promoted the decrease of NF-κB expression by inhibiting the degradation of IκB-α and inhibited the production of inflammatory factors [127]. It also led to a decrease in the levels of TNF and NO [127]; exogenous GSTP1 protein can be delivered to macrophages, inhibit the expression of iNOS and COX-2 in cells, and inhibit inflammation [128]. Additionally, in LPS-induced THP-1 cells, GSTP1 facilitates LC3-I to II conversion and p62 degradation by inhibiting the PI3K–Akt–mTOR signaling pathway, thereby preserving autophagy stability [129]. However, whether this mechanism operates similarly in the lung tissue remains unclear.

## Estrogen-metabolizing enzymes in PH

### CYPs in PH

**Fig. 5:**
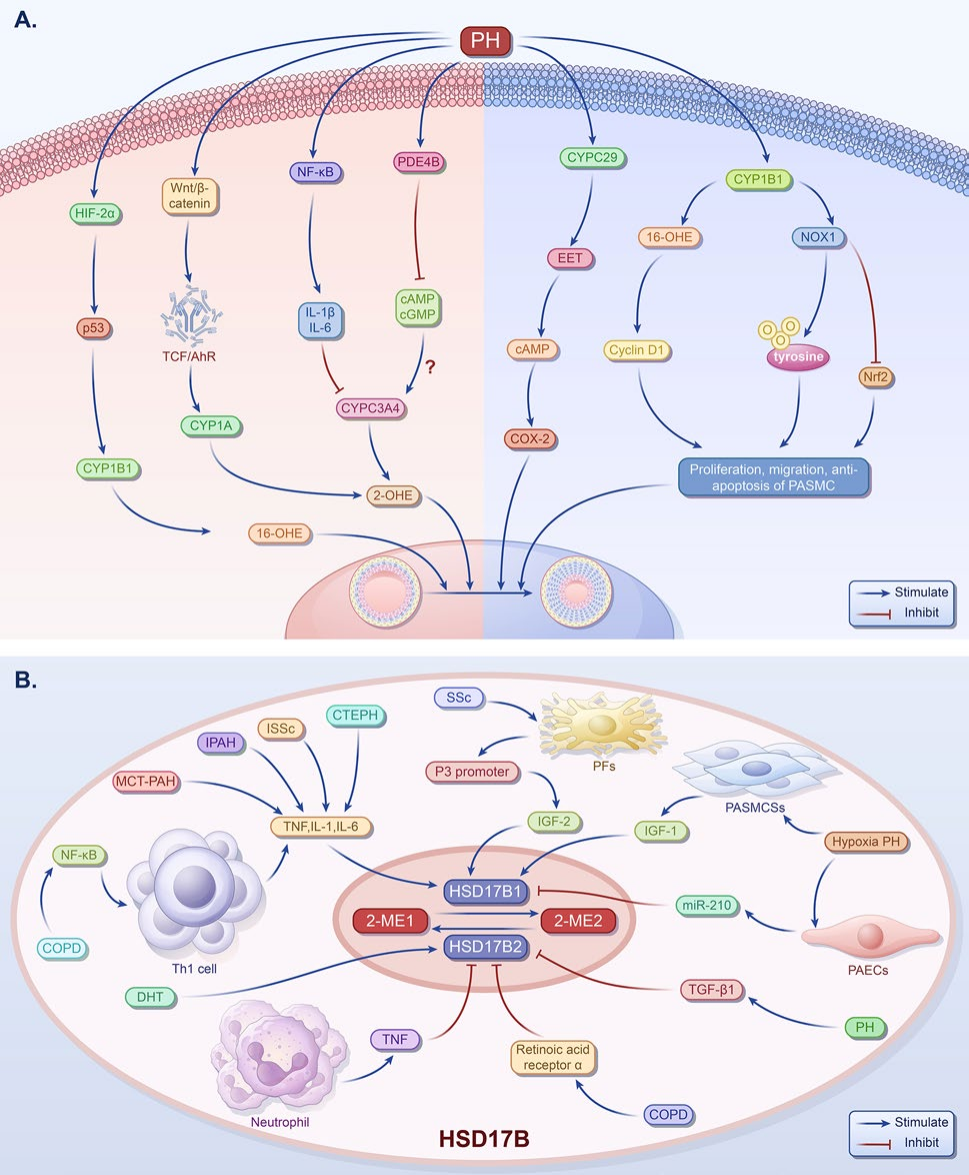
The influence factors of CYPs and HSD17B in PH. **A** The influence factors of CYPs in pulmonary hypertension. In patients with PH or animal models, the expression of HIF-2α is increased, and the upregulation of p53 may promote the expression of CYP1B1 and increase the level of 16-OHE. The increased levels of IL-1β and IL-6 may inhibit the activity of CYP3A4 and decrease the level of 2-OHE. The activation of Wnt/β-catenin signaling pathway may promote the expression of CYP1A and increase the level of 2-OHE. The expression of PDE4B is increased leads to decreased levels of cAMP and cGMP, but the content of CYP3A is not clear. The increased expression of CYP2C29 and EET increases the expression of COX-2 through a cAMP-dependent pathway, thereby promoting endothelial cell proliferation and angiogenesis. The increased expression of CYP1B1 may promote the proliferation of PASMCs by increasing the levels of 16α-OHE and cyclin D1, promoting the expression of Nox1, stimulating the irreversible oxidation of protein tyrosine phosphatase, and reducing the activity of nuclear factor Nrf2 and the expression of its antioxidant genes. **B** The influence factors of HSD17B in PH. Under PH, OSoxidative stress activates the NF-κB channel and promotes of TNF, IL-6, and IL-1 by helper T cell type 1 45, which will upregulate HSD17B1. In addition, in PH, the upregulation of HSD17B1 and the increase of HSD17B1 will increase the production of E2 and decrease the production of E1. Due to TNF, ATRA, and DHT, HSD17B2 increases, resulting in increased E1 production and decreased E2 production. HIF-2α, hypoxia-inducible factor-2alpha; CYP, cytochrome P450; 2-OHE, 2-hydroxyestradiol; PH, pulmonary artery hypertension; OS, oxidative stress; IL, interleukin; PDE4B, phosphodiesterase 4B; cAMP, cyclic adenosine monophosphate; cGMP, cyclic guanosine 3′:5′-monophosphate; EET, endoscopic eradication therapy; MAP, mitogen-activated protein; COX-2, cyclooxygenase-2; Nox1, nicotinamide adenine dinucleotide phosphate oxidase1; Nrf2, nuclear factor E2-related factor 2; NF-κB, nuclear factor-kappa B; HSD17B, 17β-hydroxysteroid dehydrogenase; TNF, tumor necrosis factor; IGF, insulin-like growth factor

CYPs are a class of cytochrome P450 enzymes mainly located in the liver, lungs, and other organs that regulate cell function and estrogen metabolism [130, 131]. Studies have indicated that a hypoxic environment can affect CYP activity in vivo. For instance, in rabbits subjected to hypoxia, the activity of CYPs initially remains relatively stable but subsequently decreases with prolonged exposure to hypoxia [132], suggesting a compensatory response followed by decompensation. Extensive studies and comprehensive analyses revealed that alterations in the expression of relevant factors or pathological processes in PH can influence the levels or activities of CYPs, thereby exacerbating or ameliorating PH progression (Fig. 5A).

#### CYPs contribute to PH through E2 metabolism

CYP1B1 is markedly expressed in pulmonary artery lesions of patients with PH, but its expression is significantly lower in the pulmonary arteries of patients without PH [39]. Studies have shown that PFTα (a p53 inhibitor) can inhibit the activity of CYP1A1, CYP1A2, and particularly CYP1B1 [133]. In the PAECs of SU5416/hypoxia-induced PH rat model, hypoxia promotes the expression of HIF-2α, leading to the upregulation of p53 [134]. Therefore, we speculate that upregulated p53 may increase the level of 16α-OHE by promoting the expression of CYP1B1 and ultimately promoting PH development. Studies have found that IL-6, TNF, interferon γ, TGF-β, and IL-1β can inhibit the expression of CYPs such as CYP3A4, CYP1A2, CYP2B6, and CYP2C8 [135]. In patients with PH, IL-1β and IL-6 levels are increased [136]. Therefore, we speculated that CYP3A4 activity is inhibited in patients with PH, resulting in a decrease in 2-OHE2 levels, thereby aggravating PH.

Wnt signaling affects the development of various diseases [137]. The Wnt/β-catenin signaling pathway is activated in PASMCs of patients with PH [138, 139]. Studies have found that Wnt/β-catenin promotes the expression of CYP1A (and other CYP subtypes) in the liver through TCF or aromatic hydrocarbon receptors (AhR) on DNA [140]. Therefore, we speculate that the increased expression of CYP1A in the PASMCs of patients with PH leads to an increase in 2-OHE2 levels, alleviating the deterioration of PH. In mouse hepatocytes, activation of PKA decreases the expression of CYP3A; however, calmodulin-dependent protein kinase and cGMP-dependent protein kinase can promote the expression of CYP3A [141]. PDE4B, a specific cyclic adenosine monophosphate (cAMP) hydrolase [142], can reduce the levels of cAMP and cGMP and is highly expressed in rat with PH [143].However, the specific effects on CYP3A levels remain unclear.

#### CYPs contribute to PH through non-E2 metabolism

Additionally, CYPs may contribute to pulmonary vascular remodeling and abnormal proliferation of pulmonary vascular smooth muscle through non-E2 metabolism, further promoting the onset and progression of PH. Pokreisz et al. found that in Swiss Webster mice subjected to long-term hypoxia, increased expression of CYP2C29 in the lung tissue led to elevated levels of EET, increasing COX-2 expression through a cAMP-dependent pathway, thereby promoting ECs proliferation and angiogenesis [144, 145] and ultimately, PH development. This effect was reduced by a selective cyclooxygenase inhibitor (*N*-methylsulfonyl-6-[2-propargyloxyphenyl] hexamide; MSPPOH) [144]. In addition, CYP1B1 is highly expressed in patients with PH and mice, and it may upregulate cyclin D1 levels by increasing the expression of mitogens such as 16α-OHE [39]; it promotes the expression of Nox1, stimulates the irreversible oxidation of protein tyrosine phosphatase, reduces the activity of Nrf2 and the expression of its antioxidant genes [42], promotes the proliferation of PASMCs, and, eventually, leads to PH deterioration.

### HSD17B in PH

HSD17B catalyzes the conversion of 17-ketones into 17-hydroxysteroids. So far, 15 HSD17B proteins have been identified [146]. HSD17Bs are polymers expressed in various organisms, with NADPH as a cofactor. The 15 members of HSD17Bs, HSD17B1 to HSD17B15, are mainly responsible for the redox reactions of hormones, fatty acids, and bile acids [147]. Among these, HSD17B1 and HSD17B2 are important in estrogen metabolism (Fig. 5B). HSD17B1 is an estrogen stage 3 metabolic enzyme that catalyzes the conversion of 2-ME1 to 2-ME2, whereas HSD17B2 has the opposite effect [148]. In addition, studies have found that the level of HSD17B1 in males is lower than in females [149]. A decrease in androgen levels may inhibit the expression of HSD17B2 [150, 151], and the overall expression of HSD17B decreases with time after menopause in women [152], suggesting a relationship between HSD17B and the PH estrogen paradox.

#### Upregulated HSD17B1 in PH

It was found that TNF, IL-1, and IL-6 can promote the expression of HSD17B1 [153, 154]. Chronic pulmonary obstruction disease (COPD), one of the causes of PH, belongs to the third PH group. Oxidative stress, as an important pathogenic factor of COPD, can activate NF-κB [155] and promote the production of inflammation-related factors TNF, IL-1, and IL-6 [156] in helper T cell type 1. At the same time, many studies have found increased expression of inflammation-related factors in patients with multiple types of PH. Zhong et al. collected hemodynamic data of idiopathic patients with PAH through right cardiac catheterization and found that the expressions of TNF, IL-1, and IL-6 were significantly higher than those of control subjects [157]. In patients with left heart failure combined with PH, the expression of the TNF inflammatory factor increased [158]. Serum expressions of TNF, IL1-β, ICAM-1, and IL-6 increased in patients with localized SSc with PH [159]. In patients with chronic thromboembolic PH (CTEPH), a type of PH caused by persistent pulmonary thromboembolism, serum levels of circulating TNF, IL-6, IL-8, and MIP-1α are high [160, 161]. In addition, increased levels of inflammatory factors have been observed in animal PH models. The expression of caspase-8 was increased in macrophages of lung tissue of monocroline-treated rats, which promoted inflammatory cell infiltration by activating the NLRP3/IL-1β signaling pathway [162]. Tang et al. found that in the rat PAH experiment induced by monocrotaline, the expression levels of TNF, IL-1, and IL-6 in rat lung tissue increased [163]. It was inferred that HSD17B1 expression is upregulated in patients with PH and animal models. In addition, insulin-like growth factor (IGF)-1 and IGF-2 upregulate HSD17B1 expression [153]. In hypoxia-induced PH-induced neonatal mouse PASMCSs, IGF-1 expression was significantly increased [164, 165]. SSc can occur in several overlapping forms of PH [166]. Some studies have shown that the IGF-2 P3 promoter is activated in a pathological model of SSc-related pulmonary fibrosis, which improves IGF-2 mRNA in SSc lung fibroblasts [167]. Therefore, in the pathological condition of PH, upregulation of TNF, IL-1, IL-6, IGF-1, and IGF-2 promote HSD17B1 expression (Fig. 5B).

#### Downregulated HSD17B1 in PH

However, in addition to cytokines, miR-210 targets HSD17B1 [168]. In the lung tissues of mice with hypoxic PH and hypoxic sugen-induced PH, miR-210 is significantly upregulated in hypoxic pulmonary ECs [169, 170]. At the same time, Huang et al. confirmed that increased miR-210 in plasma can be a potential diagnostic marker for COPD-PH [171]. Similarly, upregulated miR-210 downregulates HSD17B1 expression in PH (Fig. 5B). In summary, the specific expression of HSD17B1 in PH is unclear, and direct evidence is needed, such as the expression levels of HSD17B1 in the plasma, lung tissue, and pulmonary vessels of patients with PH.

#### HSD17B2 inhibits PH

Salama et al. demonstrated that TNF can inhibit the expression of HSD17B2 [154]. In patients with COPD, it was found that the expression of TNF in neutrophils increased [172]. Yoshiaki et al. observed reduced expression of HSD17B2 mRNA in endometrial stromal cells cultured with retinoic acid for four consecutive days [173]. However, the expression of retinoic acid receptor α in the plasma of patients with COPD is increased during disease progression [174]. In addition, HSD17B2 was downregulated in TGF-β1 treated prostate stromal cells [175]. In rats with PH induced by increased blood flow, TGF-β1 protein expression levels were increased [176]. It is inferred that TNF, retinoic acid, and TGF-β1 in the pathological condition of PH inhibit HSD17B1 expression (Fig. 5B). At the same time, it was found that DHT could induce the expression of HSD17B2 mRNA and protein in endometrial cancer cells [177]. DHT can promote monocrotaline-induced pulmonary artery remodeling and right ventricular hypertrophy and participates in the occurrence and development of PAH in male rats [178]. This suggests that males may have higher HSD17B2 levels than females.

In summary, the level of HSD17B1 in the body is uncertain in the pathological condition of PH. However, the overall level of HSD17B2 is decreased, which may lead to the conversion of 2-ME2 to 2-ME1. As reviewed above, 2-ME2 exerts a protective effect during PH development, thereby alleviating pathological processes.

## Medicine therapy targeting E2 metabolism

Anastrozole (Table 1), functioning as an aromatase inhibitor, effectively prevents the conversion of testosterone into E2, leading to reduced E2 levels [179]. In preclinical studies, administration of anastrozole significantly mitigated pulmonary vascular remodeling, decreased right ventricular systolic pressure, and reduced right ventricular hypertrophy in hypoxia-induced PH female mice compared with male mice [180]. In a larger phase II trial investigating anastrozole (PHANTOM [Pulmonary Hypertension and Anastrozole Trial]; NCT03229499), anastrozole significantly decreased E2 levels and increased 6-min-walk distance without improvement in right ventricular function and quality of life in patients with PAH [181]. In a open-label proof-of-concept pilot study, fulvestrant treatment was shown to improve right ventricular function and increase 6-min-walk distance in patients with PAH through reducing 16α-OHE2 [182]. The combination treatment of anastrozole and fulvestrant has been shown to significantly reduce the percentage of muscularized pulmonary vessels and right ventricular systolic pressure in BMPR2 mutant PAH mice [183]. Moreover, in hypoxia-induced PH model, exogenous supplementation of 2ME2 has demonstrated promising therapeutic effect [69, 70, 77]. Recently, traditional Chinese medicines targeting HSD17B and CYPs also exert beneficial effects in alleviating PH by affecting the levels of estrogen products through, such as wogonin and *Crocus sativus* L. [184, 185]. These findings further emphasize the critical role of estrogen and its metabolites in driving the progression PH (Table 1). They also reinforce the therapeutic potential of modulating estrogen metabolite levels in PH, suggesting that targeted regulation of these pathways may offer an effective strategy for disease management. This highlights estrogen metabolism as a promising avenue for future treatments aimed at improving patient outcomes in PH.

**Table 1:** Medicine therapy targeting E2 metabolism for PH

Disease (models)	Intervention	Target	Effect	References
Hypoxia-PAH rat, patients with PAH (phase 2 randomized clinical trial)	Anastrozole	Decrease E2	Decrease pulmonary vascular remodeling, decrease right ventricular systolic pressure, increase the 6 min walk distance by 26 m	[180, 181]
Patients with PAH	Fulvestrant	Decrease 16α-OHE2	Increase right ventricular systolic pressure, improve right ventricular function and increase 6 min walk distance by 31 m	[182]
BMPR2 mutant mouse	Anastrozole + fulvestrant	Decrease E2	Reduce the percentage of muscularized pulmonary vessels and right ventricular systolic pressure	[183]
Hypoxia-PH rat	2-ME2	Increase 2-ME2	Mitigate the pulmonary angiogenesis, reduce pulmonary artery remodeling, right ventricular systolic pressure, right ventricular hypertrophy and oxidative stress	[69, 70, 77]
MCT-PAH rat	Wogonin	Increase HSD17B2 and 2-ME2	Retard PAH and inhibit EndMT	[184]
Patients with COPD	*Crocus sativus* L.	Decrease HSD17B2, increase HSD17B1 and 2-ME2	Improve inflammation	[185]

## Perspective and conclusions

The estrogen 16a-OHEs metabolites have been implicated in promoting the development of PH, whereas 2-ME1 has been identified as a protective factor inhibiting PH progression. However, there is still controversy over the role of 4-OHEs. Studies conducted by Peng et al. at the Fox Chase Cancer Center in Philadelphia in 2012 and 2017 revealed significant sex disparities in estrogen metabolite levels in the lung tissues of mice and humans [186, 187]. In normal lung tissue, the absolute levels and percentages of 4-OHEs and 16a-OHEs are higher in females than in males [187]. Furthermore, sex differences in the levels of 4-OHEs and 16a-OHEs were significantly elevated in female smokers compared with males [187]. This suggests a potential link between elevated levels of harmful estrogen metabolites and increased susceptibility to PH in females. Conversely, sex differences were also noted in the levels of the protective estrogen metabolite 2-OMEs, with higher levels observed in female lung tissues, irrespective of smoking status. This may contribute to milder disease manifestations in females compared with males.

Changes in estrogen metabolism are inevitably associated with the enzymes involved in metabolism. Therefore, we summarize the current findings on the relationship between estrogen-metabolizing enzymes and PH. First, the expression and activity of estrogen phase I metabolizing enzymes, CYPs, have been linked to hypoxic exposure. However, there is currently no evidence of sex-based differences in CYPs. Previous studies have indicated that the AhR transcriptionally regulates certain CYPs. The AhR can compete with HIF-1α for binding to HIF-1β, reducing HIF-1α transcriptional activity. Docherty et al. found gender differences in HIF-1α signaling in human PASMCs [77]. It can be inferred that the expression of CYPs may exhibit gender differences through the gender-specific regulation of HIF-1α, which could be one of the reasons for the gender disparities observed in PH. However, further research is needed to confirm this hypothesis.

Second, the phase II metabolizing enzyme 2-ME1 levels, HSD17B1, were lower in males than in females. In addition, androgens stimulate the expression of HSD17B2. Therefore, in males, the predominant metabolite of 2-OMEs is the less protective compound, 2-ME1, which may contribute to the increased severity of the disease compared with that in females. Furthermore, the expression levels of HSD17B decrease in a time-dependent manner in postmenopausal women, which could be one of the reasons for the higher incidence and severity of disease in postmenopausal women. Currently, there is no direct evidence on the expression of HSD17B before and after the onset of PH nor is there any sex difference in patients with PH. Therefore, investigating sex differences and the molecular mechanisms underlying changes in estrogen-metabolizing enzymes induced by PH could provide a basis for a more comprehensive explanation of sex disparities in PH.

Based on the role of estrogen metabolites in PH, we hypothesized that in early hypoxia, a significant reduction in CYP1A1 leads to a marked decrease in 2-ME2 levels, with 16a-OHE and 4-OHE being the predominant metabolites, notably higher in females compared with males, promoting SMC proliferation and vascular remodeling and contributing to a higher disease incidence in females. Under prolonged hypoxia, CYP3A4 levels significantly decrease, leading to an increase in 2-ME2 production. Additionally, females exhibited higher HSD17B1 levels than males, resulting in a significant increase in 2-ME2 levels. Consequently, females exhibited milder disease progression than males in the mid-to-late stages. Recent studies suggested that the preventive effects of 2-ME2 are inferior to its therapeutic effects, particularly in the limited improvement of right ventricular hypertrophy [188]. Therefore, the precise utilization of estrogen metabolites in relation to the duration of hypoxia indicates that exogenous estrogen therapy may yield superior outcomes with significant clinical implications.

## Data Availability

Not applicable.

## Abbreviations

PH: Pulmonary hypertension
BMPR2: Bone morphogenetic protein receptor type II
E2: Estradiol
CYPs: Cytochrome P450s
UGTs: UDP-glucuronosyltransferases
2-OHE2: 2-Hydroxyestradiol
4-OHE2: 4-Hydroxyestradiol
2-ME2: 2-Methoxyestradiol
HSD17B: 17β-Hydroxysteroid dehydrogenase
2-ME1: 2-Methoxyestrone
16α-OHE: 16α-Hydroxyestrone
ER: Estrogen receptor
PASMCs: Pulmonary arterial smooth muscle cells
PAECs: Pulmonary arterial endothelial cells
EndoMT: Endothelial mesenchymal transition
ROS: Reactive oxygen species
Nrf2: Nuclear factor E2-related factor 2
PPARγ: Peroxisome proliferator-activated receptor γ
HIF: Hypoxia-inducible factor
SSc: Systemic sclerosis
PAH: Pulmonary arterial hypertension
ECs: Endothelial cells
TGF-β: Transforming growth factor-β
IL: Interleukin
TNF: Tumor necrosis factor
SMCs: Smooth muscle cells
HASMCs: Human aortic smooth muscle cells
GPR30: G protein-coupled receptor 30
GSTP: Glutathione *S*-transferase P
COPD: Chronic pulmonary obstruction disease
IGF: Insulin-like growth factor
